# Development of Jelly Loaded with Nanogel Containing Natural L-Dopa from *Mucuna pruriens* Seed Extract for Neuroprotection in Parkinson’s Disease

**DOI:** 10.3390/pharmaceutics14051079

**Published:** 2022-05-17

**Authors:** Chuda Chittasupho, Sarin Tadtong, Suwanna Vorarat, Witcha Imaram, Sirivan Athikomkulchai, Weerasak Samee, Vipaporn Sareedenchai, Thanu Thongnopkoon, Siriporn Okonogi, Narisa Kamkaen

**Affiliations:** 1 Department of Pharmaceutical Sciences, Faculty of Pharmacy, Chiang Mai University, Mueang, Chiang Mai 50200, Thailand; chuda.c@cmu.ac.th (C.C.); siriporn.okonogi@cmu.ac.th (S.O.); 2 Research Center of Pharmaceutical Nanotechnology, Faculty of Pharmacy, Chiang Mai University, Mueang, Chiang Mai 50200, Thailand; 3 Department of Pharmacognosy, Faculty of Pharmacy, Srinakharinwirot University, Ongkharak, Nahonnayok 26120, Thailand; sarin@g.swu.ac.th (S.T.); sirivan@g.swu.ac.th (S.A.); vipaporn@g.swu.ac.th (V.S.); 4 Department of Pharmaceutical Chemistry, Faculty of Pharmacy, Srinakharinwirot University, Ongkharak, Nahonnayok 26120, Thailand; suwannav@g.swu.ac.th (S.V.); weerasak@g.swu.ac.th (W.S.); 5 Department of Chemistry and Center of Excellence for Innovation in Chemistry, Faculty of Science, Kasetsart University, Chatuchak, Bangkok 10900, Thailand; witcha.i@ku.ac.th; 6 Department of Pharmaceutical Technology, Faculty of Pharmacy, Srinakharinwirot University, Ongkharak, Nakhonnayok 26120, Thailand; thanu@g.swu.ac.th; 7 Department of Industrial Pharmacy, School of Pharmacy, Eastern Asia University, Thanyaburi, Pathum Thani 12110, Thailand

**Keywords:** *M. pruriens* seed, functional food, Parkinson’s disease, levodopa, nanogel

## Abstract

The first line therapy of patients with Parkinson’s disease, a neurodegenerative disorder caused by the degeneration of dopaminergic neurons, is levodopa (L-dopa) given orally. Recently, the presence of natural L-dopa in the seed of *Mucuna pruriens*, a tropical legume in the Fabaceae family, was reported and it showed superior efficiency compared with synthetic L-dopa. Therefore, this study aimed to examine the phytochemical compounds, particularly for natural L-dopa, in *M. pruriens* seed extract and subsequently prepare a nanogel containing the extract prior to incorporation into a jelly formulation for use as a functional food in elderly patients with Parkinson’s disease. The results show that *M. pruriens* seed extract contains phenolic compounds, flavonoids, tannins, alkaloids, terpenoids, and saponins. The quantitative analysis performed by the HPLC method revealed that spray-dried *M. pruriens* seed extract contained 5.59 ± 0.21% L-dopa. *M. pruriens* seed extract possesses a ferric-reducing antioxidant power and shows free-radical scavenging activity, determined by *DPPH* and *ABTS* methods, suggesting a distinctive antioxidant ability of the extract. *M. pruriens* seed extract at 10 ng/mL did not show cytotoxicity against a neuronal cell line (SH-SY5Y cells), kidney cells (HEK293 cells), or Caco-2 cells. Nanogel of *M. pruriens* seed extract prepared by ionic gelation had the hydrodynamic diameter, polydispersity index and zeta potential value of 384.53 ± 11.24 nm, 0.38 ± 0.05, and −11.23 ± 1.15 mV, respectively. The transepithelial transport of L-dopa in *M. pruriens* seed-extract nanogel through Caco-2 cells was measured. Nanogel containing *M. pruriens* seed extract at the concentration of 10 ng/mL exhibited neuroprotective activity. A jelly formulation containing *M. pruriens* seed-extract nanogel was successfully developed. The prepared jelly exhibited the acceptable physical and microbiological stabilities upon 6 months of the stability test. The half-life of natural L-dopa in jelly were 3.2, 0.9, and 0.6 years for storage conditions at 4, 30, and 40 °C, respectively, indicating the thermal degradation of natural L-dopa. The prepared jelly containing natural L-dopa from *M. pruriens* seed extract with the prominent antioxidant activity is a promising option for elderly patients suffering from Parkinson’s disease.

## 1. Introduction

*Mucuna pruriens* (*M. pruriens*) is a tropical legume in Fabaceae family. Mucuna seed contains abundant natural L-dopa, which has been shown to be more tolerated and more potent than synthetic L-dopa [1,2,3]. Pre-clinical and clinical studies have shown that *M. pruriens* seed containing natural L-dopa had benefits over synthetic L-dopa in terms of higher efficacy and lower adverse effect. Katzenschlager et al. reported the faster onset of action and longer duration of *M. pruriens* seed powder formulation without concomitant increase in dyskinesias, suggesting that this natural source of L-dopa might possess advantages over conventional synthetic L-dopa in the long-term management of Parkinson’s disease [4]. Hussian et al. revealed the efficacy of *M. pruriens* seed extract as an anti-Parkinson’s agent in the intrastriatal 6-OHDA parkinsonian animal model. They also showed that *M. pruriens* seed extract was more effective than synthetic L-dopa, whereas a pharmacokinetic profile of *M. pruriens* seed extract was similar to the combination of L-dopa and carbidopa [5]. *M. pruriens* seed extract did not show acute or sub-chronic toxicity in mice and rabbits [6]. In a Parkinson’s Disease Study Group, HP-200 derived from *M. pruriens*, was shown to have efficacy and tolerability in patients with Parkinson’s disease; it significantly reduced the Hoehn and Yahr stage and UPDRS scores, and only produced mild adverse effects. It was hypothesized that *M. pruriens* seed extract may contain several compounds that have anti-Parkinson activity in addition to L-dopa [7]. Lieu et al. reported that *M. pruriens* endocarp powder + carbidopa and synthetic L-dopa + carbidopa ameliorated parkinsonism in monkeys. However, synthetic L-dopa + carbidopa treatments significantly increased substantia nigra reticulata bursting firing patterns that were not seen with *M. pruriens* endocarp powder + carbidopa treatments. They also showed that oral *M. pruriens* seed-water extract improved parkinsonism without causing drug-induced dyskinesia. *M. pruriens* acted through a different mechanism from that of synthetic L-dopa, according to a distinctive neurophysiological finding in the basal ganglia [8]. In another study, *M. pruriens* seed powder provided significantly more behavioral benefit compared with synthetic L-dopa and reduced the severity of drug-induced dyskinesia. The authors suggested that *M. pruriens* seed-water extract contained water soluble ingredients that have dopa-decarboxylase inhibitor-like activity [9]. *M. pruriens* seed extract in a pharmaceutical formulation may serve as a dietary supplement to delay the need to increase the L-dopa dose, delay the combination therapy of L-dopa, and accelerate the onset of L-dopa activity [10].

*M. pruriens* seed extract is now marketed in the form of capsules. The problem with using *M. pruriens* seed extract is the unknown concentration of L-dopa in the capsule. The high concentration of L-dopa in the *M. pruriens* seed extract may lead to severe adverse effects or toxicity, particularly when co-administration with anti-Parkinson’s drugs. Therefore, the identification, quantification, and absorption of the bioactive substances, especially L-dopa, in *M. pruriens* seed extract are necessary for determining the dosage regimen of this extract in Parkinson’s patients. Generally, L-dopa is available in tablets and capsules administered orally. The recommended dose is 300 to 1200 mg/day, divided into three to twelve doses [11]. Elderly Parkinson’s patients are found to have difficulty in swallowing tablets and capsules, which affects drug compliance. In this study, *M. pruriens* seed extract was used as a bioactive pharmaceutical ingredient in jelly to help geriatric patients with difficulty swallowing or infusion problems associated with gastro-intestinal tubes. However, the stability of L-dopa is a great concern in pharmaceutical product development. At room temperature and at uncontrolled pH, L-dopa levels significantly declines in a 48-h period. Therefore, in addition to developing a product that is acceptable to patients, it is also necessary to investigate the stability profile and shelf-life of the product. The factors affecting the failure of product development are poor water solubility, poor bioavailability, and rapid chemical degradation. To address these problems, encapsulation of *M. pruriens* seed extract containing L-dopa in nanogel is proposed. In this study, the nanogel was formulated to entrap the *M. pruriens* seed extract containing L-dopa and loaded in easy swallowing jelly. The nanogel and jelly were characterized. The physical, chemical, and microbiological stability of the jelly were also investigated.

## 2. Materials

L-dopa, cell proliferation kit II (XTT), cell proliferation reagent WST-1, gallic acid, *ABTS* (2,20-azino-bis(3-ethylbenzothiazoline-6-sulfonic acid)), *DPPH* (2,2-diphenyl-1-picrylhydrazyl), and TPTZ (2,4,6-Tris(2-pyridyl)-s-triazine were purchased from Sigma-Aldrich (St. Louis, MO, USA). Quercetin and epigallocatechin (EGCG) (98% purity) were purchased from Chanjao Longevity Co., Ltd. (Bangkok, Thailand). Folin–Ciocalteu phenol reagent, aluminum chloride, ferrous sulfate heptahydrate, potassium persulfate, and sodium acetate trihydrate were purchased from Loba Chemie (Mumbai, India). Minimum Essential Medium Eagle (MEM), Eagle’s Minimum Essential Medium (EMEM), Dulbecco’s Modified Eagle Medium (DMEM), and Dulbecco’s phosphate-buffered saline (DPBS) were purchased from ATCC (Manassas, VA, USA). Fetal bovine serum (FBS), antibiotic-antimyocin, F12 medium, and 0.25% trypsin-EDTA were purchased from Gibco (Canada). Aspartame, citric acid monohydrate, sodium citrate, carrageenan, ascorbic acid, sodium benzoate, and tamarind flavoring agent were purchased from Namsiang, Bangkok, Thailand.

## 3. Methods

### 3.1. M. pruriens Seed Identification, Extraction and Spray Drying

Seeds of *M. pruriens* (L.) DC. were collected by a community enterprise in the Western Forest Complex, Thailand. The morphological characteristics were identified using the taxonomic key of Mucuna plants in Thailand, by Assoc. Prof. Dr. Narisa Kamkaen, Board Certified of the College of Herbal Pharmacy of Thailand (BCHP). The voucher specimens were deposited in the medicinal plant herbarium at the School of Pharmacy, Eastern Asia University (EAU). The voucher number of the sample was EAU-MP-001. *M. pruriens* seeds (50 kg) were washed, dried, roasted, and ground to fine powders. The obtained powders (48 kg) were then boiled in water (300 L) at 100 °C with continuous stirring [12]. The obtained aqueous extract was concentrated by boiling until the soluble solid reached 8° Brix. The extract was mixed with maltodextrin (2%) as a solid carrier prior to spray drying. The mixture was dried using a spray dryer (Model SD-03-Gas). The inlet and outlet temperatures were 200 and 100 °C, respectively. The feed rate was set at 50 L/h with a total spray drying time of 5 h.

### 3.2. Phytochemical Screening of M. pruriens Seed Extract

The spray-dried extract was used for qualitative phytochemical screening for compounds, including tannins, flavonoids, alkaloids, saponins, and steroids following the described methods [13].

#### 3.2.1. Flavonoids Test 

The presence of flavonoids in *M. pruriens* seed extract was determined by the Shinoda test. Crude extract of *M. pruriens* seed (0.5 g) was dissolved in 10 mL of 95% ethanol by heating followed by filtration. The filtrate was concentrated until the final volume was 2 mL. Five fragments of magnesium ribbon and concentrated hydrochloric acid (1 mL) were mixed with the ethanolic extract. The appearance of a red, pink, or orange color revealed flavonoid was contained in the extract.

#### 3.2.2. Tannins and Phenolics Test

Crude extract of *M. pruriens* seed (0.5 g) was boiled in 10 mL of water. A quantity of 5 mL of extract was then mixed with 100 µL of 2% *w*/*v* gelatin solution and lead acetate saturated solution. Precipitation was observed, indicating the presence of tannins. Ferric chloride (1%) solution was added. The presence of phenolics was shown by a dark green or blue color.

#### 3.2.3. Alkaloids Test

Crude extract of *M. pruriens* seed (0.5 g) was stirred with 1% HCl (20 mL) in a water bath for 5 min prior to filtration. The filtrate was divided into 2 parts (2 mL for each part). Dragendorff’s reagent and Mayer’s reagent were added to each part. The presence of alkaloids in the extract was shown by the formation of white-yellow and orange precipitation in Mayer’s reagent and Dragendorff’s reagent, respectively [14].

#### 3.2.4. Terpenoids Test

Crude extract of *M. pruriens* seed (0.5 g) was mixed with dichloromethane (2 mL), and 98% sulfuric acid was slowly added to make a layer. The reddish-brown color ring appeared on the sulfuric acid and dichloromethane interface to show the presence of terpenoids in the extract.

#### 3.2.5. Saponins Test

Crude extract of *M. pruriens* seed (0.5 g) was mixed with 5 mL of distilled water in a test tube and shaken. Persistent froth was observed after being shaken vigorously and the mixture was left for 20 min [14].

### 3.3. Quantitative Analysis of Phenolic Compounds and Total Flavonoid Content in M. pruriens Seed Extract

Total phenolic content in *M. pruriens* spray dried seed extract was determined by the Folin and Ciocalteu reaction [15]. Gallic acid and *M. pruriens* seed extract were dissolved in deionized water at concentrations of 3.9–2000 µg/mL. The solutions of gallic acid and *M. pruriens* extract were added into 96-well plates (50 µL/well). Folin and Ciocalteu’s phenol reagent (10% *v*/*v*, 100 µL) was added and mixed for 1 min. Na_2_CO_3_ solution (10% *w*/*v*, 50 µL) was added to the mixture after 4 min of incubation. The solution was left for 2 h at room temperature and its absorbance was measured with a UV–Visible spectrophotometer (Spectramax M3, Thermo Scientific, Waltham, MA, USA) at 765 nm. The total phenolic contents were calculated as µg gallic acid equivalents per mL of crude extract and µg/mg gallic acid equivalents of crude extract by using the gallic acid calibration curve.

The total flavonoid content in spray dried *M. pruriens* extract was determined by the aluminum chloride colorimetric method [15]. Quercetin, epigallocatechin (EGCG), and extract solution at concentrations of 3.9–2000 µg/mL (100 µL/well) were added with NaNO_2_ (5%, 30 µL) in 96-well plates. After 5 min of incubation, aluminum chloride (2% *w*/*v*, 50 µL) was added and incubated for 6 min followed by 10 min incubation with NaOH (1 N, 50 µL). The absorbance of the mixture was measured at 510 nm with a UV–Vis spectrophotometer (Spectramax M3, Thermo Scientific, Waltham, MA, USA). The total flavonoid contents were calculated by using quercetin and EGCG calibration curves. Data were expressed as µg quercetin and EGCG equivalents per mL of crude extract and µg/mg quercetin and EGCG equivalents of crude extract.

### 3.4. Chemical Analysis of L-Dopa in M. pruriens Seed Extract

#### 3.4.1. Thin Layer Chromatography

Thin layer chromatography (TLC) was used to separate and determine the presence of *L-dopa* in *M. pruriens* seed extract. The mobile phase was modified from the previous method [16]. Silica gel GF254 (Merck, Darmstadt, Germany) was used as the stationary phase and a mixture of butanol, methanol, acetic acid, and water (12.0:6.5:2.5:1.5) was used as the mobile phase. *L-dopa* (1 mg/mL, 5 µL) and spray-dried *M. pruriens* seed extract (2 mg/mL, 10 µL) were spotted on the stationary phase using Camag^®^ Linomat 5 (Dublin, Ireland). After development, the plates were sprayed with 2% Ninhydrin solution. The plate was then photographed under visible light, 254, and 366 nm using the TLC visualizer. The original TLC images were analyzed by the winCATs TLC workstation program (Camag, Ireland).

#### 3.4.2. High Performance Liquid Chromatography

Analysis was performed by an HPLC system equipped with a UV detector (Thermo Separation Product, Waltham, MA, USA). Chromatographic separation was carried out using C_18_ column (Inertsil^®^ ODS-3, 250 mm × 4.6 mm, i.d. 5 µm) with isocratic elution and UV detection at 283 nm [17]. The mobile phase consisted of a 0.025 M ortho-phosphoric acid buffer pH 2.5 and pumped at a flow rate of 1.0 mL/min. The standard solution containing 20, 40, 60, 80, and 100 µg/mL of *L-dopa* was prepared in 0.1 N HCl. For the assay, an accurate weight of spray-dried *M. pruriens* seed extract powder was transferred into 25 mL-volumetric flasks. An amount of 15 mL of 0.1 N HCl was then added, and the solution was sonicated in an ultrasonic bath for 20 min and adjusted to volume with 0.1 N HCl. The prepared solutions were then filtered through 0.45 μm filters and the first 1 mL of the filtrate was discarded. The filtrates were injected into the column.

### 3.5. Determination of M. pruriens Seed Extract Antioxidant Activity

#### 3.5.1. *DPPH* Free-Radical Scavenging Activity

The *DPPH* free-radical scavenging capacity of *M. pruriens* seed extract was determined and compared to gallic acid [15]. Gallic acid standard solution and *M. pruriens* seed extract sample solution were prepared by dissolving compound and extract in deionized water at concentrations ranging from 3.9 to 2000 µg/mL. *DPPH* at a concentration of 500 µM dissolved in the absolute ethanol (100 µL) was mixed with the gallic acid and *M. pruriens* seed extract solution (100 µL) and incubated at room temperature for 30 min. A UV–Vis spectrophotometer microplate reader was used to measure the absorbance of the reactants at a wavelength of 517 nm. The *DPPH* radical scavenging activity was calculated by Equation (1). The IC_50_ values of gallic acid and *M. pruriens* extract were calculated from the non-linear regression analysis by GraphPad Prism 7.02 software.

(1) $$DPPH\;Free\;radical\;scavenging\;\left(\%\right)=\left(\frac{A-B}{A}\right)\times 100\%$$

where *A* is the absorbance of the reaction with solvent control and *B* is the absorbance of the reaction with samples.

#### 3.5.2. *ABTS* Free-Radical Scavenging Activity

The standard gallic acid solution and *M. pruriens* seed extract solution (3.9–2000 µg/mL) were prepared in deionized water. The *ABTS* scavenging assay was performed using the method in a previous report [18]. *ABTS* radical cation (*ABTS*^+^) was generated by reacting *ABTS* stock solution (7 mM) with potassium persulfate (2.45 mM) for 24 h in a dark at room temperature. Before use, *ABTS*^+^ radical cation was diluted with absolute ethanol to obtain an optimal absorbance of 0.70 (±0.02) at 734 nm. The standard and sample solutions (20 µL) were mixed with *ABTS*^+^ free radicals (180 µL). The mixture was then incubated at room temperature for 15 min. The absorbance at a wavelength of 734 nm was measured by a microplate reader. The *ABTS*^+^ radical scavenging activity was calculated by Equation (2). The IC_50_ values of gallic acid and *M. pruriens* extract were calculated from the non-linear regression analysis by GraphPad Prism 7.02 software.

(2) $$ABTS\;Free\;radical\;scavenging\;\left(\%\right)=\left(\frac{A-B}{A}\right)\times 100\%$$

where *A* is the absorbance of the reaction with solvent control (deionized water) and *B* is the absorbance of the reaction with the extract.

#### 3.5.3. Ferric Reducing Antioxidant Power

Gallic acid and *M. pruriens* seed extract solutions (3.9–2000 µg/mL) were prepared in deionized water. A FRAP reagent was prepared by mixing an acetate buffer (300 mM, pH 3.6) with 10 mM TPTZ in 40 mM HCl, and 20 mM FeCl_3_ at 10:1:1 (*v*/*v*) ratio. The gallic acid and sample solutions (20 µL) were reacted with the FRAP reagent (180 µL) for 30 min at 37 °C [15]. The absorbance was then measured at a wavelength of 595 nm. The standard curve of the ferrous sulfate solution (9.8–2500 μM) was constructed to calculate the μmol Fe (II) equivalent of the gallic acid and sample. 

### 3.6. Preparation and Characterization of M. pruriens Seed-Extract Nanogel

*M. pruriens* seed-extract nanogel was prepared based on the ionic gelation of hyaluronic acid (HA) and magnesium chloride (MgCl_2_). HA was dissolved in deionized water to form a 2 mg/mL HA solution. *M. pruriens* seed extract was dissolved in deionized water (0.2 g/mL) and the extract solution (3 mL) was mixed with the HA solution (2 mL). To prepare *M. pruriens* seed-extract nanogel, *M. pruriens* seed extract and HA mixture were placed on the magnetic stirrer stirring at 900 rpm, 1 mL of MgCl_2_ solution (0.2 mg/mL) was dropwise added to the extract–HA solution using a syringe pump at a flow rate of 3 mL/min. The reaction was carried out for 10 min and the resulting nanogel was subjected to further analysis.

*M. pruriens* seed-extract nanogel was characterized for size, size distribution, and surface charge. The hydrodynamic diameter and polydispersity index of the nanogel were determined using a Zetasizer Nano ZS (Malvern Instruments, Worcestershire, UK) based on dynamic light scattering. The zeta potential of the nanogel was measured by electrophoretic light scattering using the Zetasizer Nano ZS. For the stability study, *M. pruriens* seed-extract nanogel in deionized water, containing *M. pruriens* seed extract 100 mg/mL, was kept in a vial and stored at 4 °C, 30 °C, and 45 °C for 6 months. At predetermined time intervals i.e., 0, 0.5, 1, 2, 3, 4, 5, and 6 months, the sizes, polydispersity index (PDI), and zeta potentials of the nanogel were determined using dynamic light scattering technique.

The encapsulation efficiency of the nanogel was determined by quantitative analysis of *L-dopa* using the HPLC method mentioned in Section 3.4.2. The accurate weight of jelly containing *M. pruriens* seed-extract nanogel was transferred into 25 mL volumetric flasks. An amount of 15 mL of 0.1 N HCl was then added, and the solution was sonicated in an ultrasonic bath for 20 min and adjusted to volume with 0.1 N HCl. Samples were analyzed by HPLC. The encapsulation efficiency of *L-dopa* in the nanogel is described by the following equation.

(3) $$Encapsulation\;efficiency\;\left(\%\right)=\frac{Amount\;of\;L-dopa\;in\;the\;nanogel}{Amount\;of\;L-dopa\;in\;the\;extract}\times 100\%$$

The in vitro release study was performed by transferring the nanogel (10 mL) to a dialysis bag suspended in the release media (500 mL), namely simulated gastric fluid (SGF) pH 1.2, simulated intestinal fluid (SIF) pH 6.8, and phosphate-buffered saline (PBS) pH 7.4. The SGF was composed of sodium chloride (2% *w*/*v*) and concentrated hydrochloric acid (7% *w*/*v*) in purified water. The SIF was prepared by dissolving potassium dihydrogen phosphate (6.8% *w*/*v*) and sodium hydroxide (0.9% *w*/*v*) in purified water. The PBS was purchased from Gibco. Subsequent released samples were taken at pre-determined intervals i.e., 0.5, 1, 2, 3, 4, 6, and 8 h by removing 1 mL of media and replacing with 1 mL of fresh media to prevent saturation of *L-dopa*. The solubility of *L-dopa* in water was about 5 mg/mL; therefore, it could be proposed that the sink condition in the release system could be maintained. The amount of *L-dopa* released was quantified by UV–Visible spectrophotometry analysis at a wavelength of 283 nm and the release percentage was calculated by Equation (4).

(4) $$Drug\;release\;\left(\%\right)=\frac{Amount\;of\;L-dopa\;in\;the\;medium}{Amount\;of\;L-dopa\;in\;the\;nanogel}\times 100\%$$

The sensitivity of UV–Visible spectrophotometry was justified by the limit of detection (LOD) and limit of qualification (LOQ) of this assay. The LOD was calculated from the formula 3.3(σ/slope) and the LOQ was calculated from the formula 10(σ/slope), where σ and slope are the standard deviations of the *y*-axis intercept and slope of a standard curve, respectively.

### 3.7. Neuroprotective Assay of M. pruriens Seed Extract and Nanogel against SH-SY5Y Cells

The human neuroblastoma cell line SH-SY5Y ATCC CRL-2266 was obtained from ATCC (Manassas, VA) and maintained in EMEM: F12 supplemented with 10% FBS and 1% antibiotic–antimycotic solution at 37 °C in 5% CO_2_/95% air. SH-SY5Y cells were treated with 0.1, 0.2, 0.4, 0.8, and 1.0 mM MPP^+^ and the cell viability was measured by XTT assay [19]. The highest concentration of MPP^+^ that resulted in the greatest toxicity to SH-SY5Y cells was further used for neuroprotective study. The cytotoxicity of 0.001–10 ng/mL *L-dopa* and 0.01–100 ng/mL *M. pruriens* seed extract was investigated by XTT assay. For the neuroprotective assay, SH-SY5Y cells were incubated with MPP+ and co-incubated with the highest non-cytotoxic concentration of *L-dopa* and *M. pruriens* seed extract for 24 h. Samples were removed, and the cell viability was determined by XTT assay. XTT solution (50 µL of 1 mg/mL XTT in culture medium and 25 µM phenazine methosulfate) was added to the cells and incubated at 37 °C for 4 h. After incubation, PBS (100 µL) was added, and the absorbance value was read using a microplate reader at 450 nm.

### 3.8. Cytotoxicity Assay of M. pruriens Seed Extract and Nanogel against HEK293 Cells

The human embryonic kidney cell line HEK293 ATCC CRL-1573 was obtained from ATCC (Manassas, VA, USA) and maintained in EMEM supplemented with 10% FBS and 1% antibiotic–antimycotic solution at 37 °C in 5% CO_2_/95% air [20]. The cytotoxicity of 0.01–1000 µg/mL *L-dopa*, 0.01–100 µg/mL *M. pruriens* seed extract, and 0.02–5000 µg/mL nanogel were investigated by XTT assay using the above-mentioned method [20].

### 3.9. Permeability Study of M. pruriens Seed-Extract Nanogel across Caco-2 Cells

Caco-2 cells were cultured and maintained in Dulbecco’s Modified Eagle Medium (DMEM) supplemented with 10% fetal bovine serum (FBS) and 1% penicillin–streptomycin at 37 °C, 5% CO_2_. Caco-2 cells (2 × 10^5^ cells/well) were seeded in 96-well plates and incubated for 24 h at 37 °C, 5% CO_2_. *M. pruriens* seed-extract nanogel was suspended in DMEM (100 µL) at various concentrations (0.001–100 mg/mL). The nanogel was added to the cells and incubated at 37 °C in 5% CO_2_ for 24 h. After incubation, the nanogel was removed, and the cells were washed with PBS. WST-1 (10% *v*/*v* of DMEM) was added to the cells (100 µL/well) and incubated for 30 min at 37 °C in 5% CO_2_. The absorbance was then measured at 450 nm. The cell viability percentage was calculated using Equation (5), where the negative control was the viability of untreated cells. The positive control used in this assay was mitomycin C (20 µg/mL). The IC_50_ value was calculated based on non-linear regression analysis.

(5) $$Cell\;viability\;\left(\%\right)=\frac{A450\;of\;tested\;cells}{A450\;of\;control}\times 100\%$$

The cellular morphology of Caco-2 cells was investigated using an inverted microscope (EVOS, Thermo Fisher Scientific, Waltham, MA, USA). The change of cellular morphology indicating cell death was scored using the criteria of International standard ISO 10993-5. The scores of 0, 1, 2, 3, and 4 indicated no toxic, slightly toxic, mildly toxic, moderately toxic, and severely toxic reactivity, respectively [21].

Caco-2 cells were seeded on Transwell polyester membranes (Corning, Somerville, MA, USA) at a density of 2 × 10^4^ cells/well in DMEM containing 15% FBS and 1% penicillin–streptomycin and cultured for 21 days to form monolayers. The integrity of the cell monolayers was assessed by measuring their Transepithelial electrical resistance (TEER) value every other day during experiments. *M. pruriens* seed-extract nanogel was added to a cell monolayer at 1 and 10 mg/mL (200 µL/well) and TEER values were measured at 0, 30, 90, 180, and 360 min. After TEER value measurements, the medium in the outer chamber was collected for analysis of the *L-dopa* permeated concentrations at each time point. The transport concentration of the sample was analyzed using LC/MS/MS and the concentration of permeated *L-dopa* at each time point was calculated according to the respective standard curve. For liquid chromatography, separation was performed at 40 °C using an ExionLC™ AD system, with a Phenomenex 2.6 μm C18 (100 × 4.6 mm). An elution gradient of ultrapure water/0.5% (*v*/*v*) formic acid and methanol/0.5% (*v*/*v*) formic acid was used over 7 min, using a flow rate of 0.3 mL/min with a 5 μL injection volume. MS and MS/MS data were collected using MRM^HR^ acquisition on the SCIEX X500R QTOF system using the electrospray ionization (ESI) probe run in positive ion mode, using ionspray voltage of 5500 V at 450 °C. TOF MS (scan range: 100–300 Da) parameters were 80 V declustering potential, 10 V collision energy, and 0.25 sec accumulation time. The mass transitions were observed at m/z of 198→152.1017 and quantitative analysis was performed using SCIEX OS software (SCIEX).

### 3.10. Development of Jelly Containing M. pruriens Seed-Extract Nanogel

#### 3.10.1. Formulation of Jelly Containing *M. pruriens* Seed-Extract Nanogel

Aspartame (0.2% *w*/*w*), citric acid monohydrate (1% *w*/*w*), and sodium citrate (0.5% *w*/*w*) were dissolved in hot water (46.5% *w*/*w*). *M. pruriens* seed-extract nanogel (50% *w*/*w*) and carrageenan (0.5% *w/w*) were added into the above solution and homogeneously mixed. When the temperature of the mixture was 40 °C, ascorbic acid (1% *w/w*), sodium benzoate (0.2% *w/w*), and tamarind flavoring agent were added. The jelly was filled and sealed in an aluminum-foil air-tight container and kept in the refrigerator for 20 min before further analysis.

#### 3.10.2. Physical and Chemical Stabilities of Jelly Containing *M. pruriens* Seed-Extract Nanogel

To investigate the effects of temperature and time on the physical and chemical stability of jelly containing *M. pruriens* seed-extract nanogel, samples were kept at 4 °C, 30 °C, and 40 °C for 6 months. The rheology and viscosity of the jelly were investigated at 0, 1, 2, 3, 4, 5, and 6 months using a Thermo Scientific HAAK E RheoStress 1 rheometer equipped with a plate and plate geometry (1.0 mm gap, 60 mm diameter). The rheological behavior and viscosity profiles of the samples were presented at a shear rate ranging from 0.1 to 200 s^−1^ [22]. The pH values of jelly stored at 4, 30, and 40 °C were determined using a pH meter (Model 340, Mettler Toledo GmbH, Hessen, Germany). The remaining amount of *L-dopa* in jelly containing *M. pruriens* seed-extract nanogel was quantified by HPLC analysis using the method mentioned in Section 3.4.2.

#### 3.10.3. Microbiological Stability of Jelly Containing *M. pruriens* Seed-Extract Nanogel

Counts for Coliform and *E. coli*, total aerobic bacteria, yeast and fungi, Staphylococcus, and Salmonella were determined according to manufacturer’s instructions [23]. The jelly containing *M. pruriens* seed-extract nanogel freshly prepared and stored at 4, 30, and 40 °C (1 mL) was diluted (1:1000) with sterile buffered peptone water and dispersed onto the center of Compact Dry™ EC, Compact Dry™ TC, Compact Dry™ YM, Compact Dry^TM^ X-SA, and 3M Petrifilm^TM^ Salmonella Express (SALX). The plates were sealed and incubated at 37 °C for 24 h. For detecting yeast and mold contamination, samples were diluted and placed on a 3M^TM^ Petrifilm^TM^ Yeast and Mold count plate. The plate was incubated at 25 °C for 96 h. All Compact Dry and 3M Petrifilm^TM^ count plates were observed and compared to a negative control, which was a sterile buffered peptone solution.

### 3.11. Statistical Analysis

Statistical analysis of all experiments was performed by an analysis of variance (one-way ANOVA), followed by Tukey as a post-hoc test. In all cases, a value of *p* < 0.05 was considered statistically significant. All data represent mean ± S.D. with triplicate experiments.

## 4. Results and Discussion

### 4.1. Yield of M. pruriens Seed Extraction

From 50 kg of raw *M. pruriens* seed, 48 kg of roasted and cooked seeds were extracted and spray dried. The yield obtained from extraction and spray drying was 14.03 kg of dry extract, accounting for 28.06% *w*/*w*.

### 4.2. Phytochemical Compounds in Spray-Dried M. pruriens Seed Extract

*M. pruriens* seed extract contained several types of phytochemical constituents, including phenolic compounds, flavonoids, tannins, alkaloids, terpenoids, and saponins. *M. pruriens* seed was shown to contain several nutritional and pharmacological compounds, including *L-dopa* which is the first-line treatment for Parkinson’s disease [24]. It was reported that *L-dopa* derived from *M. pruriens* has many advantages over synthetic *L-dopa* when administered to Parkinson’s patients, since synthetic *L-dopa* can have several side effects when used for many years [25]. Although *M. pruriens* seed contains *L-dopa* as a major compound, other bioactive phytochemical compounds were reported, including phenolic compounds, flavonoids, tannins, saponins, carbohydrates, and proteins [26]. In this study, we found that phytochemical compounds of *M. pruriens* seed extract presented different types of secondary metabolites. The phytochemical screening assay clearly shows that *M. pruriens* seed extract contains phenolic compounds, flavonoids, tannins, alkaloids, terpenoids, and saponins. Phenolic compounds are known as potential antioxidants by acting as radical scavengers. Flavonoids are a class of plant secondary phenolics with significant antioxidant and chelating properties. Tannins are a polyphenolic compound acting as antioxidant and free-radical scavenging agents to reduce oxidative stress. Two tetrahydroquinoline alkaloids, namely (-) 3-methoxy-1,1-dimethyl-6,7- dihydroxy-1,2,3.4-tetrahydroquinoline and (-) 3- methoxy-1,1-dimethyl-7,8-dihydroxy-1,2,3.4- tetrahydroquinoline, have been found in *M. pruriens* seed extract [27]. Most alkaloids often have pharmacological effects and are used as medications. Shanmugavel and Krishnamoorthy reported the presence of terpenoids and saponins in *M. pruriens* seed extract [28].

### 4.3. Total Phenolic and Total Flavonoid Contents in Spray-Dried M. pruriens Seed Extract

**Figure 1:**
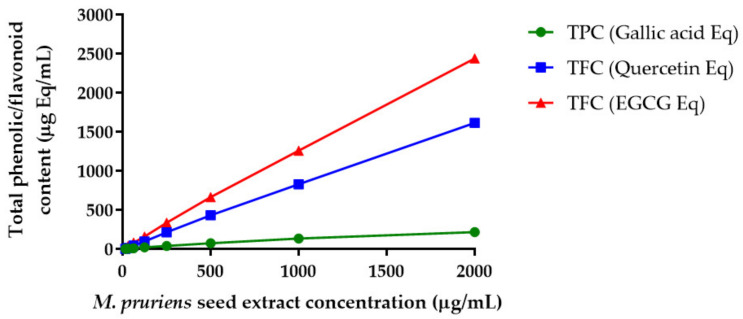
Total phenolic content (TPC, gallic acid equivalent) and total flavonoid content (TFC, quercetin or epigallocatechin equivalent) of *M. pruriens* seed extract.

Phenolic compounds are bioactive constituents responsible for antioxidant activity due to the hydroxyl groups of the phenolic compounds that can scavenge free radicals. The results were derived from a calibration curve (y = 0.0129x + 0.0763, R^2^ = 0.9995) for gallic acid (4–125 µg/mL) and expressed in gallic acid equivalents (GAE) per gram dry extract weight (Figure 1). The content of phenolic compounds in *M. pruriens* seed extract ranged from 81.2 to 160.6 mg GAE/g dry crude extract. The flavonoid contents in spray-dried *M. pruriens* seed extract were determined using the aluminum chloride method. The content of flavonoids in the extract was calculated from the calibration curve (y = 0.0012x + 0.0533, R^2^ = 0.9997) for quercetin (4–2000 µg/mL) and expressed in quercetin equivalents (QE) per gram extract weight and EGCG equivalents per gram extract. The flavonoid contents in *M. pruriens* seed extract ranged from 48 to 807 mg QE/g and from 1080 to 1219 mg EGCG equivalent/g dry crude extract.

The total phenolic and flavonoid contents in the *M. pruriens* extract observed in this study were higher than those in previous reports. The *M. pruriens* growing location, extract solvent, and extraction method affected the yield of bioactive compounds in the seed extract. Longhi et al. reported that total phenolic content in their acid extract of *M. pruriens* was 240 mg/g of extract [29]. In another study, Sisshuraju et al. demonstrated the undesirable effect of heat on the total phenolic contents in *M. pruriens* extract. The total phenolic content in the dry heat and moist heat-treated *M. pruriens* seed significantly reduced from 62.3 mg/g extract to 32.4 and 24.3 mg/g extract, respectively [30]. Jimoh et al. reported that *M. pruriens* seed aqueous extract contained 12 mg GAE/g extract and 3.18 mg QE/g extract [31]. Theansungnoen et al. reported that the total phenolic compounds in Thai mucuna seed extract was 21.78 ± 3.58 mg GAE/g extract and total flavonoid content found in this plant seed was 423.48 ± 6.71 mg QE/g extract [32].

### 4.4. Qualitative and Quantitative Analysis of L-Dopa in M. pruriens Seed Extract

#### 4.4.1. TLC Analysis of *L-Dopa* in *M. pruriens* Seed Extract

The qualitative analysis of *L-dopa* in spray-dried *M. pruriens* seed extract was examined by TLC analysis. *L-dopa* in spray-dried *M. pruriens* seed extract was well separated and the spots were clearly observed at 254, 366 nm and with ninhydrin spray (Rf = 0.51). The position of the spots on the plate obtained from *M. pruriens* seed extract (5 and 10 µL) and *L-dopa* standard solution suggest that *L-dopa* is present in *M. pruriens* seed extract. The dark spots appearing on the bright background under UV 254 nm detection suggest that *L-dopa* in *M. pruriens* seed extract is a UV-active compound containing chromophores that undergoes fluorescence quenching. At UV 366 nm, dark and light blue spots were shown when *M. pruriens* seed extract was separated on the TLC plate. These results suggest that the extract might contain conjugated aromatic compounds, such as phenolic compounds and flavonoids.

#### 4.4.2. HPLC Analysis of *L-Dopa* in *M. pruriens* Seed Extract

The HPLC peak and retention time of *L-dopa* in nanogel was shown at 7.66 min, suggesting that the HPLC assay method developed in this study was capable of separating *L-dopa* from other compounds and degradation products. Thus, the developed method was selective and can be used for the analysis of *L-dopa* concentration in samples, including nanogel and jelly. Linear detection at 283 nm was observed in the *L-dopa* concentration range of 20–100 µg/mL with a correlation coefficient (R^2^) value of 1. Relative standard deviation (RSD) values were less than 1 for all the injected concentrations. The quantitative analysis performed by the HPLC method revealed that spray-dried *M. pruriens* seed extract contained 5.59 ± 0.21% *L-dopa*.

### 4.5. Antioxidant Activity of M. pruriens Seed Extract

**Figure 2:**
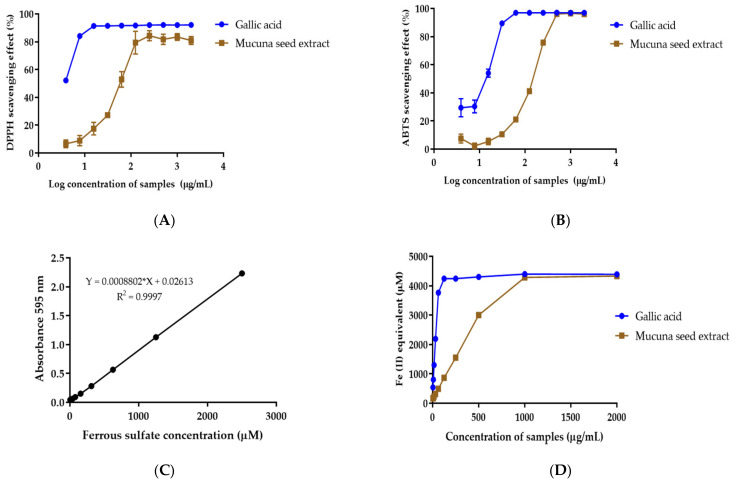
Antioxidant activity of gallic acid and *M. pruriens* seed extract determined by (**A**) *DPPH* free-radical scavenging assay, (**B**) *ABTS* free-radical scavenging assay, (**C**) standard curve of ferric reducing antioxidant power assay using ferrous sulfate, (**D**) FRAP assay.

*M. pruriens* seed extract shows *DPPH* free-radical scavenging activity with an IC_50_ value of 61.02 µg/mL (Figure 2A). The IC_50_ value of gallic acid used as a positive control was 3.39 µg/mL. *M. pruriens* seed extract shows the scavenging activity of the *ABTS*^+^ radical to have an IC_50_ value of 137.00 µg/mL (Figure 2B). The IC_50_ value of gallic acid was 11.31 µg/mL.

A FRAP assay was performed to determine the reducing capacity of *M. pruriens* seed extract in a redox reaction. The results reveal good linearity of ferrous sulfate obtained within the range of 9.8–2500 µM (R^2^ = 0.9999) (Figure 2C). The results of the FRAP assay were expressed as Fe^2+^ equivalent. Gallic acid and *M. pruriens* seed extract at 1 mg/mL exhibited 4877.13 ± 34.43 µM and 4778.25 ± 56.20 µM Fe^2+^ equivalent, respectively (Figure 2D). The results indicate the potency of *M. pruriens* seed extract to reduce ferric ion.

*M. pruriens* seed extract shows antioxidant activities by free-radical scavenging and ferric-reducing activity. Polyphenolic compounds, including phenolic acid and flavonoids, are known to have antioxidant activity, which might be one of the mechanisms involved in the neuroprotective property of *M. pruriens* extract after such phenolic compounds cross the blood–brain barrier and accumulate in the brain region. Oxidative stress due to free radicals is a leading causes of dopaminergic neurodegeneration in the substantia nigra, which results in Parkinson’s disease. Oxidative stress in the brain increases membrane peroxidation and DNA damage. The increase in iron in substantia nigra was also found in Parkinson’s disease patients. The ability of the extract to reduce ferric ions may be another neuroprotective mechanism as it reduces the lipid peroxidation in the brain tissue. The results for free-radical scavenging and the ferric-reducing power of *M. pruriens* seed extract suggest that the extract could prevent radical-induced oxidative damage, which may reduce the progress of the disease in Parkinson’s patient [33,34].

### 4.6. Particle Size, Size Distribution, Zeta Potential, and Drug Release of M. pruriens Seed-Extract Nanogel

**Figure 3:**
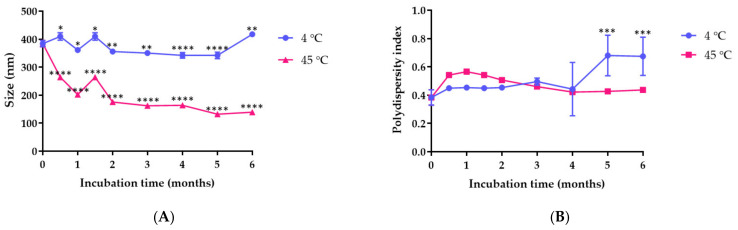

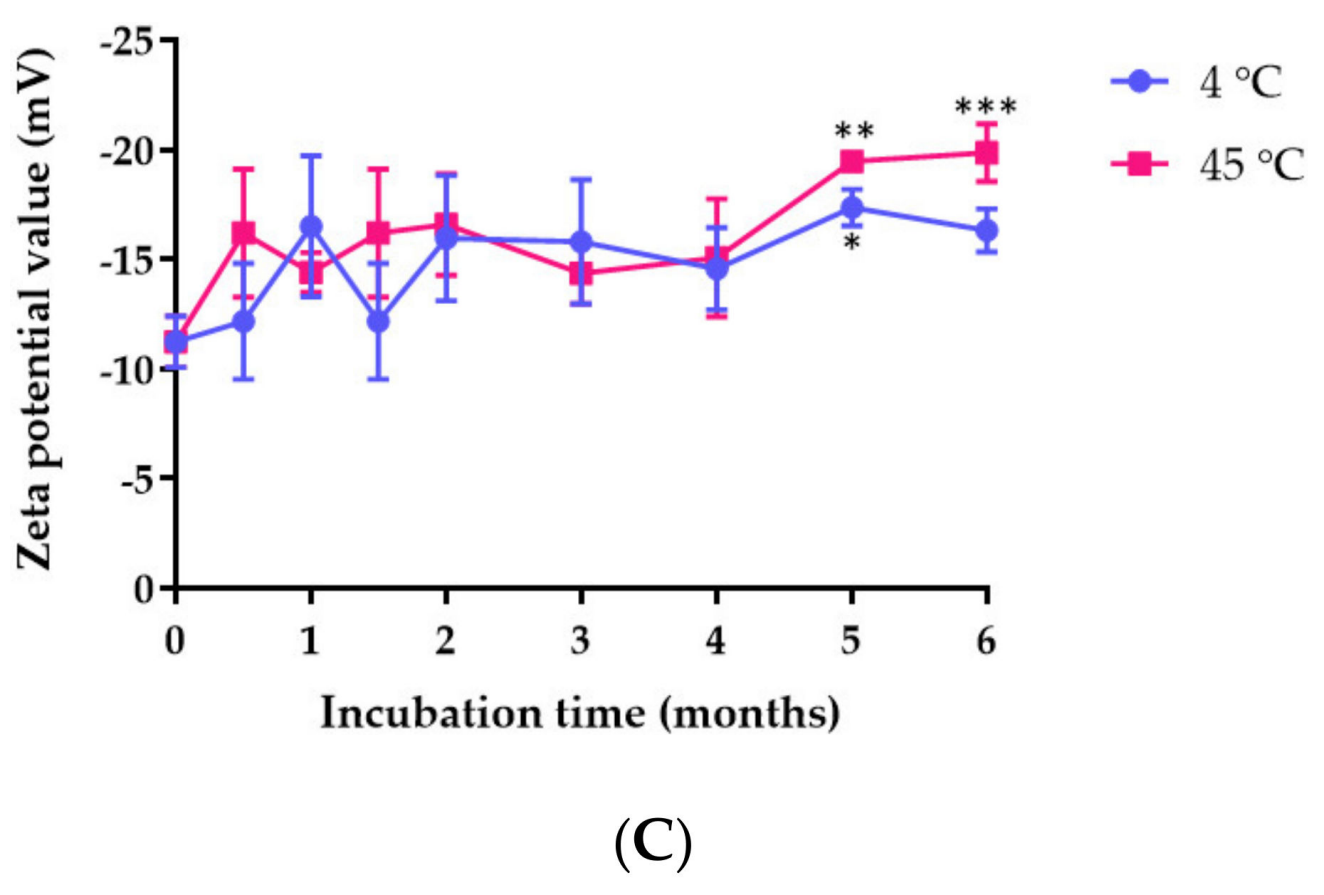
Effect of time and temperature on (**A**) particle size, (**B**) PDI, and (**C**) zeta potential of *M. pruriens* seed-extract nanogel. Data are presented as the mean ± SD. *, **, ***, and **** indicate *p* < 0.05, *p* < 0.01, *p* < 0.001, and *p* < 0.0001 compared to day 0, respectively.

Self-assembled nanogel was prepared using ionic gelation with a negatively charged molecule i.e., hyaluronic acid and a positively charged molecule i.e., magnesium chloride as a cross-linking agent. The nanogel for the *M. pruriens* seed extract was created by an electrostatic interaction between HA and MgCl_2_. The average particle size of *M. pruriens* seed-extract nanogel after freshly preparation was 384.53 ± 11.24 nm. The polydispersity index and zeta potential values were 0.38 ± 0.05 and −11.23 ± 1.15 mV, respectively. To demonstrate the colloidal stability of *M. pruriens* seed-extract nanogel, the hydrodynamic diameter, PDI, and zeta potential values of the nanogel were measured after preparation and storage at 4 °C and 45 °C. *M. pruriens* seed-extract nanogel was more stable when stored at 4 °C. The nanogel stored at 45 °C decreased in size over time (Figure 3A). The polydispersity index of *M. pruriens* seed-extract nanogel ranged from 0.3 to 0.49 and from 0.38 to 0.56 when stored for 4 months at 4 °C and 45 °C, respectively (Figure 3B). The PDI of the nanogel increased to 0.68 after 6-month storage at 4 °C, indicating physical instability of the nanogel. The zeta potential of *M. pruriens* seed-extract nanogel did not significantly change when stored at 4 °C for 6 months, but became significantly more negatively charged when stored at 45 °C for 6 months (Figure 3C). The results suggest that *M. pruriens* seed-extract nanogel should be kept at 4 °C for up to 4 months. The HPLC data show that the initial concentration of *L-dopa* in nanogel was 0.1535 mg per 100 mg of nanogel, accounting for an encapsulation efficiency of 103.00 ± 0.15%.

Nanogel is defined as a colloidal hydrogel entrapping active compounds in the network of a hydrophilic polymer. Nanogels are soluble in water and can absorb a high amount of water. The space in the network within the swollen nanogel is responsible for high drug-loading capacity. The self-assembling of HA and MgCl_2_ was constructed from the non-covalent interactions involving inter- and intramolecular cross-linking of HA, where hydroxyl groups of HA mediated hydrogen bonding and carboxyl groups take part in electrostatic interaction with Mg^2+^ ion [35]. The nanogel entrapped *M. pruriens* seed extract through electrostatic interaction, hydrophobic interaction, and π-π stacking to help solubilize the drug and protect it from chemical and enzymatic degradation [35]. Hyaluronic acid (HA) is a ligand of CD44 which is a key molecule in regulating blood–brain barrier integrity. Hence, using HA as a component of nanogel may increase blood–brain barrier permeability [36].

**Figure 4:**
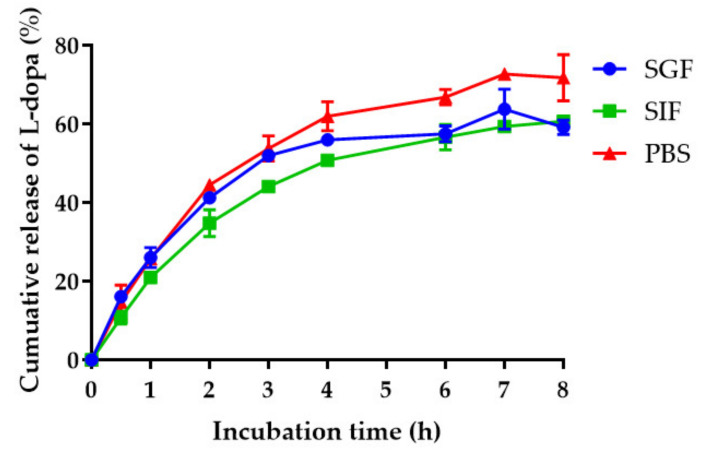
The release profile of *L-dopa* from *M. pruriens* seed-extract nanogel performed in SGF, SIF, and PBS at 37 °C. Data are presented as Mean ± SD.

The *L-dopa* release profiles of nanogel in SGF, SIF, and PBS were measured during an 8-h period at 37 °C. As shown in Figure 4, the *L-dopa* release patterns in different media are similar. The cumulative amount of *L-dopa* released in PBS was significantly higher than that of SGF and SIF, indicating pH-dependent release behavior. Different types and concentrations of counter ions in the release medium can also affect the release rate, depending on the affinity for ion-exchange with MgCl_2_ [37]. The release of *L-dopa* from the nanogel is due to hyaluronic degradation and elevated water absorption through hydrolysis [38]. Hyaluronic acid can absorb a large amount of water and expand to form a loose hydrated network [39]. Biphasic drug-release behavior was observed with an initially higher release rate in the first 4 h followed by slow and sustained release. The higher release rate is due to the hydrophilic nature of hyaluronic acid. Both diffusion and dissociation of the nanogel can control the overall release rate.

The sensitivity of UV–Visible spectrophotometry used to determine cumulative drug release was lower than that of HPLC. This method should be used to detect or quantify the concentrations of *L-dopa* that are higher than its limit of detection (LOD) and limit of qualification (LOQ) (Supplementary Table S1). In addition, the concentration of *L-dopa* could be obtained when it was in the range fitting a linear regression model (Supplementary Figure S1). The UV–Vis spectra of *L-dopa* in each medium are shown in Supplementary Figure S2.

### 4.7. Neuroprotective Effect of M. pruriens Seed Extract and Nanogel on SH-SY5Y Cells

**Figure 5:**
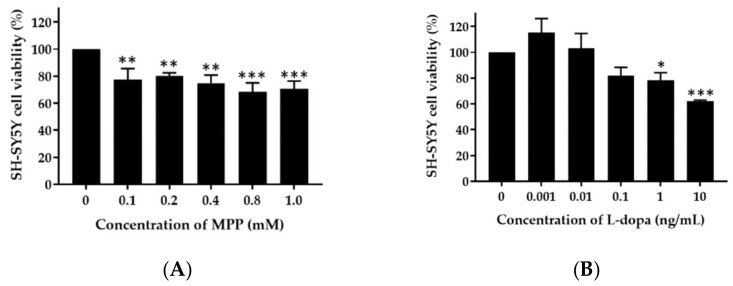

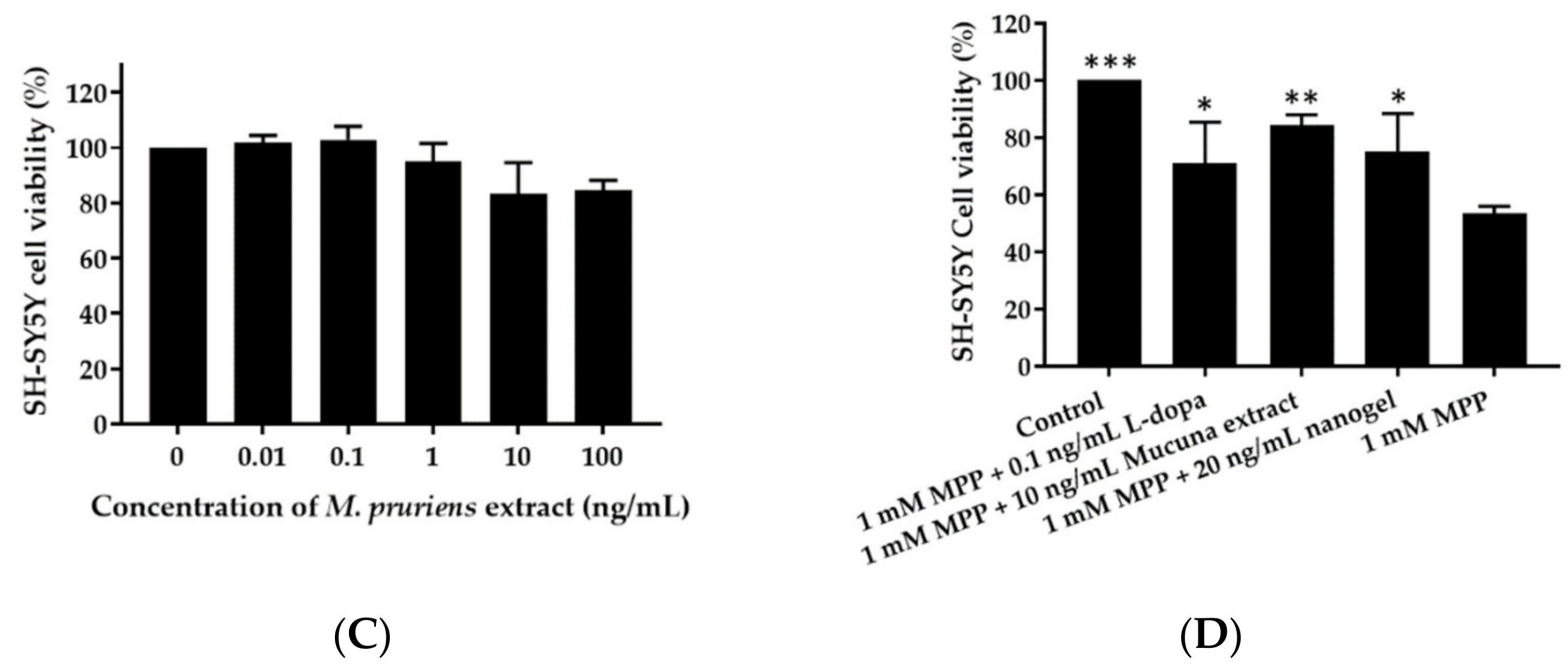
Viability of SH-SY5Y neuronal cells treated with various concentrations of (**A**) MPP^+^ (**B**) *L-dopa*, (**C**) *M. pruriens* seed extract, and (**D**) neuroprotective effect of *L-dopa*, *M. pruriens* seed extract, and nanogel containing the same concentration of tested *M. pruriens* seed extract, against SH-SY5Y cells pre-treated with MPP^+^. Data are presented as the mean ± SD. *, **, and *** indicate *p* < 0.05, *p* < 0.01, and *p* < 0.001, respectively.

SH-SY5Y neuronal cells were treated with various concentrations of MPP^+^ (0, 0.1, 0.2, 0.4, 0.8, 1.0 mM) for 24 h. At 1.0 mM, MPP^+^ reduced the cell viability to 70.7%. Therefore, a concentration of 1.0 mM MPP^+^ was selected to test the protective effect of *L-dopa* and *M. pruriens* seed extract. SH-SY5Y cells treated with 0.1 ng/mL *L-dopa* and 10 ng/mL *M. pruriens* seed extract had a cell viability of 82.03 ± 6.3% and 83.41 ± 11.0%, respectively. At these concentrations, the extract, nanogel, and *L-dopa* were investigated for their neuroprotective activity. As shown in Figure 5, the viability of SH-SY5Y cells treated with 1 mM MPP^+^ for 24 h was 53.44 ± 2.62% of the control value. Cell viability significantly increased to 71.09 ± 14.4%, 84.35 ± 3.67%, and 75.03 ± 13.41% after co-incubation with 0.1 ng/mL *L-dopa*, 10 ng/mL *M. pruriens* seed extract, and 20 ng/mL of nanogel containing 10 ng/mL *M. pruriens* seed extract. These results indicate that *L-dopa*, *M. pruriens* seed extract, and nanogel at non-toxic concentrations were able to protect SH-SY5Y neuronal cells from the cytotoxicity effect of MPP^+^.

*L-dopa*, *M. pruriens* seed extract and nanogel, at non-cytotoxic concentrations, show neuroprotective activities against SH-SY5Y cell line. The neuroprotective properties of *M. pruriens* are probably due to its antioxidant and chelating activities. Rai et al. showed that treating mice with *M. pruriens* seed can significantly reduce inflammatory parameters and prevent apoptosis of the dopaminergic neurons [40]. In addition, *M. pruriens* exhibited significant antioxidant defense by inhibiting the lipid peroxidation and nitrile level in nigrostriatal region of the mouse brain. *M. pruriens* also recovered the behavioral abnormalities in 1-methyl-4-phenyl-1,2,3,6-tetrahydropyridine (MPTP) treated mice. Additionally, *M. pruriens* treatment considerably increased the immunoreactivity of tyrosine hydroxylase and the dopamine transporter in substantia nigra pars compacta in parkinsonian mice. *L-dopa*, gallic acid, phytic acid, quercetin, and catechin equivalents were suggested as the major components which might cause neuroprotection in Parkinson’s- diseased mice [40]. Clinical study results showed that *M. pruriens* seed powder significantly reduced the Hoehn and Yahr stage and Unified Parkinson’s Disease Rating Scale (UPDRS) scores in patients from baseline to the end of 12-week period, without adverse effects [10].

### 4.8. Cytotoxic Effect of M. pruriens Seed Extract and Nanogel on Renal Cells

**Figure 6:**
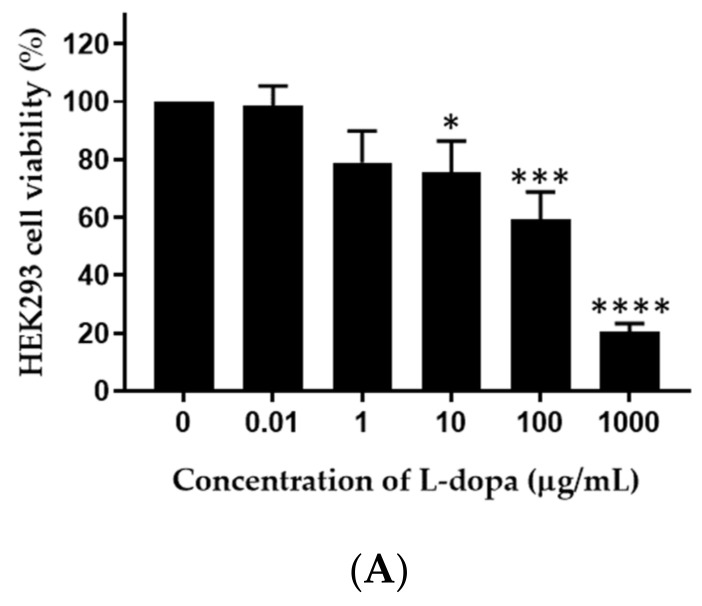

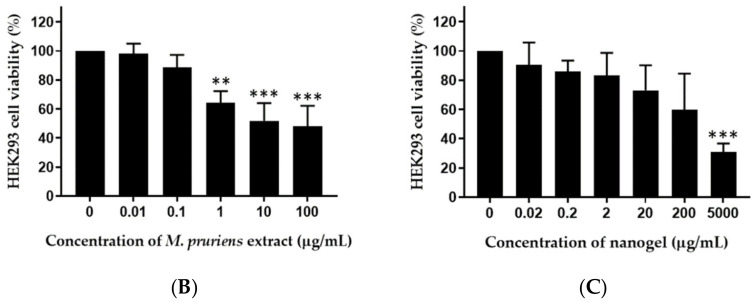
Viability of HEK293 cells after the treatment with various concentrations of (A) *L-dopa*, (B) *M. pruriens* seed extract, and (C) nanogel containing *M. pruriens* seed extract. Data are presented as the mean ± SD. *, **, ***, and **** indicate *p* < 0.05, *p* < 0.01, *p* < 0.001, and *p* < 0.0001, respectively.

The cytotoxicity of various concentrations of *L-dopa*, *M. pruriens* seed extract, and nanogel on HEK293 cell viability was investigated (Figure 6). The viability of HEK293 cells treated with 1 µg/mL *L-dopa*, 0.1 µg/mL of *M. pruriens* extract, and 0.2 µg/mL nanogel were 78.9, 88.6, and 86.1%, respectively, suggesting the non-toxic concentrations of these samples against renal cells. 

### 4.9. Permeability of M. pruriens Seed-Extract Nanogel through Caco-2 Cell Monolayer

**Figure 7:**
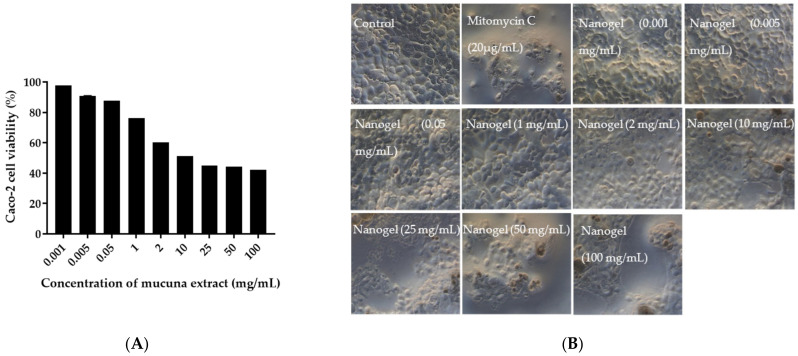
(**A**) Effect of *M. pruriens* seed-extract nanogel on the Caco-2 cell viability after incubation for 24 h. (**B**) Changes in the morphology and density of the Caco-2 cells after 24 h of exposure to various concentrations of *M. pruriens* seed extract nanogel.

The safety of *M. pruriens* seed-extract nanogel was evaluated by determining the biocompatibility and tolerability of the nanogel against Caco-2 cells used as a permeability model. A dose-dependent decrease in Caco-2 cell viability was observed after treatment with *M. pruriens* seed-extract nanogel for 24 h (Figure 7). The increase in the concentration of nanogel decreased the cell metabolic activity of Caco-2 cell line in a concentration dependent manner. At concentrations below 1 mg/mL, *M. pruriens* seed-extract nanogel did not affect Caco-2 cell viability. The IC_50_ value of nanogel against Caco-2 cells was 18.79 mg/mL. The reduction in cell viability after exposure to a high concentration of nanogel was confirmed by observing Caco-2 cell density. At a concentration of 2 mg/mL, there was a decrease in the number of cells after treatment with the nanogel. The results show that *M. pruriens* seed-extract nanogel did not exhibit cytotoxicity up to a concentration of 1 mg/mL. These results suggest the biocompatibility of *M. pruriens* extract nanogel. We, therefore, selected 1 mg/mL of *M. pruriens* seed extract in nanogel to further investigate the permeability of nanogel.

**Figure 8:**
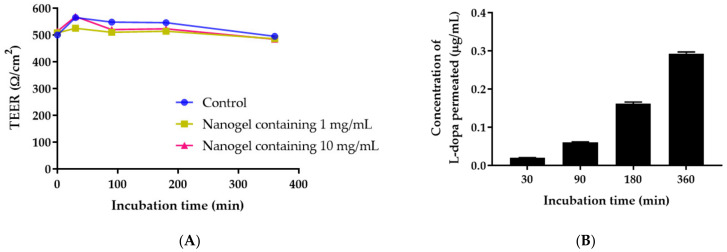
(**A**) Transepithelial electrical resistance (TEER) values of Caco-2 cell monolayer after treating the cells with *M. pruriens* seed-extract nanogel and (**B**) Concentration of *L-dopa* permeated through Caco-2 cell monolayer vs. incubation time.

TEER measurement was conducted to indicate the tightness of intercellular junction. The permeability of *M. pruriens* seed-extract nanogel was determined when TEER reached a steady state of about 500 Ω/cm^2^. As shown in Figure 8A, TEER values remained constant after Caco-2 cells were exposed to nanogel containing 1.0 and 10 mg/mL of *M. pruriens* seed extract, indicating that Caco-2 cells kept their tight junction integrity for up to 360 min. This result indicates that the permeability of *M. pruriens* seed-extract nanogel followed the normal cellular transportation process. The transepithelial transport of *M. pruriens* seed-extract nanogel was measured by determining the *L-dopa* concentration in the basolateral solution. The concentration of *L-dopa* that permeated to the basolateral side of Caco-2 cells increased with incubation time (Figure 8B). The non-saturable and linear relationship between the incubation time and the permeated concentration of *L-dopa* suggests that the transport was not involved with receptor-mediated endocytosis.

The pre-assay integrity of the Caco-2 monolayer was assessed by measuring transepithelial electrical resistance (TEER) values. The Caco-2 monolayer integrity was shown to have a TEER value of 500 Ω/cm^2^ at the steady state, indicating the strong tight junction between cells and acceptable monolayer integrity [41]. The apparent permeability was 0.32 × 10^−6^ cm/s which was considered to be low permeability. This result agrees with a previous report showing that the permeability of *L-dopa* through Caco-2 cells was very low (0.82 × 10^−6^ cm/s) [42]. It was reported that the permeability of *L-dopa* in the human intestine was 1.5 to 273-fold higher than in Caco-2 cells [42]. *L-dopa* was thought to be absorbed by amino acid transporters and the slower transport of *L-dopa* for the large neutral amino acid carrier was probably explained by lower expression compared to in vivo and saturation of the carrier [30,42,43]. Caco-2 cells were used as in vitro models for the study of intestinal dopaminergic physiology, although this may not reflect the transport phenomenon in intact tissue [44]. Siddhuraju et al. indicated that prediction of human active drug transport in Caco-2 monolayers will only be possible after characterization of each transport system and subsequent introduction of a scaling factor to compensate for the different expression of the carrier in Caco-2 cells from in vivo [30].

### 4.10. Characterization and Stability of M. pruriens Seed Extract and Jelly Containing M. pruriens Seed Extract Nanogel

**Figure 9:**
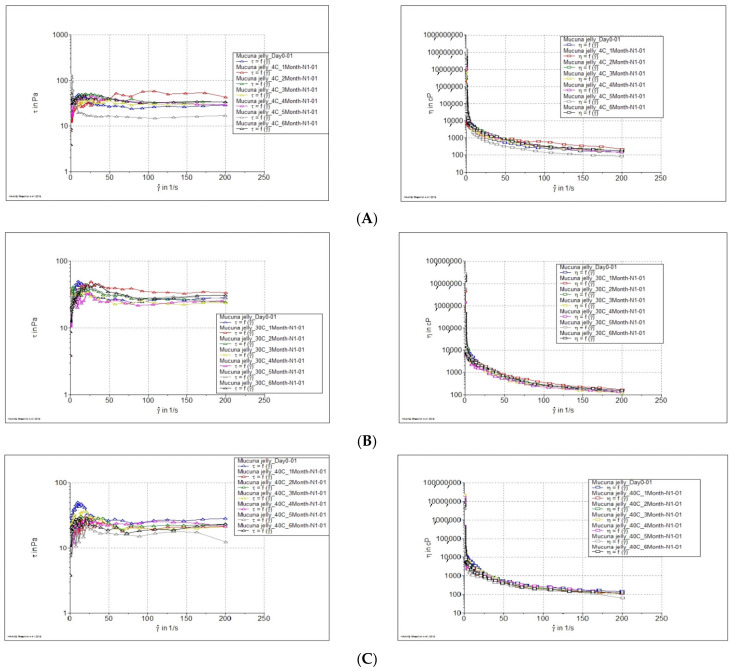

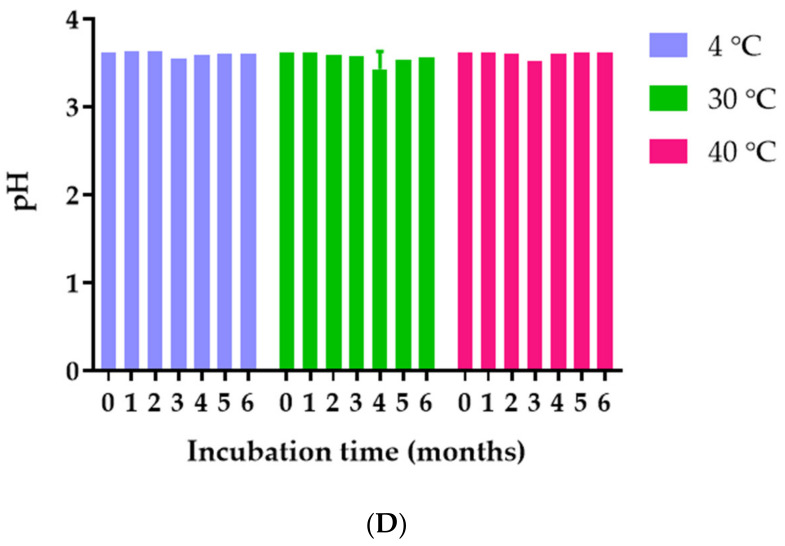
Effect of storage temperature on the viscosity (left) and rheology (right) of jelly loaded with *M. Pruriens* seed-extract nanogel as a function of shear rate after storage at (**A**) 4 °C (**B**) 30 °C, and (**C**) 40 °C for 6 months. (**D**) pH of jelly loaded with *M. pruriens* seed-extract nanogel after 6-month storage at 4 °C, 30 °C, and 40 °C.

Plots of viscosity versus shear rate for jelly containing *M. pruriens* seed-extract nanogel are shown in Figure 9A–C. The apparent viscosity of the jelly decreased significantly with increasing shear rate. In addition, the flowing behavior of the jelly occurred after the applied stress reached a critical value (yield value). These results indicate that the jelly exhibits a plastic flow behavior. The viscosity of the jelly after long-term stability (6 months) suggests that temperatures did not affect the rheology behavior of the jelly. However, the viscosity of jelly containing *M. pruriens* seed-extract nanogel decreased with increased temperature. At a shear rate of 100 (1/s), the viscosity of the jelly stored at 4, 30, and 40 °C were 292 ± 47, 237 ± 30, and 181 ± 11 cPs, respectively. The pH of the jelly was in the range of 3.43–3.63 after being freshly prepared and stored for 6 months. The results show that temperature did not affect the pH of the product (Figure 9D).

**Figure 10:**
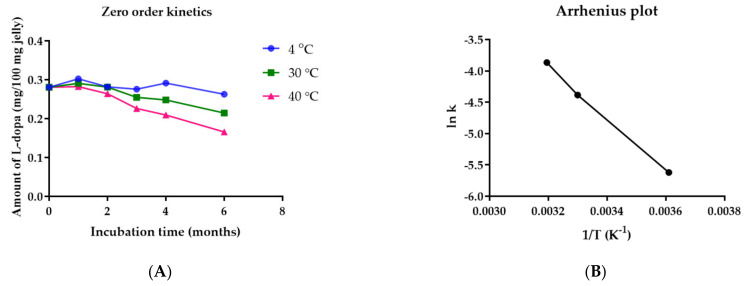
(**A**) Zero order kinetic plot of *L-dopa* amount in jelly and incubation time (**B**) Arrhenius plot of *L-dopa* in jelly containing nanogel.

The concentrations of *L-dopa* in jelly containing *M. pruriens* seed-extract nanogel stored under 4, 30, and 40 °C for 0, 1, 2, 3, 4, 5, and 6 months were analyzed. The stability results show that the quantity of *L-dopa* in the jelly reduced gradually over a period of 6 months. The concentration of *L-dopa* in jelly kept at 4, 30, and 40 °C reduced to 94.23%, 76.87% and 59.45% within 6 months from the initial 100% concentration. The linear regression analysis suggests that *L-dopa* degradation in jelly was a zero-order kinetic as the plot of the amount of *L-dopa* against incubation time yielded a straight line with R^2^ of 0.3330, 0.8925, and 0.9591 for degradation at 4, 30, and 40 °C, respectively (Figure 10A). The rate constant increased with increasing storage temperature as shown in the Arrhenius plot (Figure 10B). These results indicate that the half-life of jelly containing *M. pruriens* seed-extract nanogel depends on the storage temperature. The half-lives of *L-dopa* in jelly were 3.2, 0.9, and 0.6 years at a storage temperature of 4, 30, and 40 °C, respectively (Table 1). It is recommended that jelly containing *M. pruriens* seed-extract nanogel be stored at 4 °C to prevent chemical degradation.

The stability study shows temperature- and time-dependent *L-dopa* degradation. Pappert et al. reported that storing levodopa/carbidopa solution at a lower temperature in the presence of ascorbate could protect the drugs from degradation [45]. The degradation reaction of *L-dopa* was oxidation which was accelerated by high pH and thermal increment [46,47]. Pulikkalpura et al. reported that the degradation products of *L-dopa* degradation were dopamine, dopachrome, leucodopachrome, dopaquinone, and other reactive oxidative species [48]. Jelly developed in our study was darker in color when stored at room temperature and at 40 °C. The color of the jelly was slightly darker when stored at 4 °C. These results agree with the previous report demonstrating that the degradation rates of *L-dopa* in *M. pruriens* extract and standard *L-dopa* were low at 4 °C, compared to room temperature [48]. The high level of *L-dopa* degradation, in its pure form and in *M. pruriens* extract, into damaging quinones and reactive oxygen species requires attention since the degradation products of *L-dopa* can cause cytotoxicity in neuronal cells [48]. The jelly containing *M. pruriens* seed extract was stable at pH 3–4. The jelly exhibited plastic flow behavior with a yield point. Therefore, after the stress was no longer applied, the jelly would not flow back to its original configuration. The viscosity decreased at higher shear stress. The jelly product developed in this study shows stable physical and microbiological stability indicating an appropriate formulation. A hundred grams of jelly containing *M. pruriens* seed-extract nanogel contained 1.25 g of *L-dopa*. According to the dosage of *L-dopa* for treating Parkinson’s disease, the recommended dose of jelly was 24 g containing 300 mg of *L-dopa*. Patients with Parkinson’s disease can take 24–96 g per day to receive 300–1200 mg of *L-dopa* to achieve clinical effect. Microbiological test results show that jelly containing *M. pruriens* seed-extract nanogel stored at 4, 30, and 40 °C for 6 months were not contaminated with Coliform and *E. coli*, total aerobic bacteria, yeast and fungi, Staphylococcus, and Salmonella, indicating microbiological stability of the product.

## 5. Conclusions

In this study, we reported the non-toxic concentrations of natural *L-dopa* from *M. pruriens* seed extract against a neuronal cell line, intestinal cell line and kidney cell line. *M. pruriens* seed extract and nanogel possessed a neuroprotective effect. A nanogel of *M. pruriens* seed extract was incorporated into easy-swallowing jelly to enhance stability and delivery efficiency and to aid swallowing of the extract by elderly Parkinson’s patients. Jelly loaded with *M. pruriens* seed-extract nanogel was successfully developed. The physical and chemical stabilities of the jelly were time and temperature dependent. Jelly had a microbiological stability after storage for 6 months. Thus, this study may provide a new approach to the delivery of natural *L-dopa* from *M. pruriens* seed extract as a functional food.

## Figures and Tables

**Table 1:** Rate constant, correlation coefficient (r^2^), activation energy and half-life of *L-dopa* in jelly containing *M. pruriens* seed-extract nanogel.

k	Temperature (°C)	1/T (K^−1^)	r^2^	Ln k	Activation Energy (kJ/mol)	Half-Life (Months)	Half-Life (Years)
0.003624	4	0.00361	0.3330	−5.62018	34,670.151	38.48	3.2
0.01247	30	0.00330	0.8925	−4.38443		11.18	0.9
0.02095	40	0.00320	0.9591	−3.86562		6.66	0.6

## Supplementary Materials

The following supporting information can be downloaded at: https://www.mdpi.com/article/10.3390/pharmaceutics14051079/s1. Table S1. LOD and LOQ of *L-dopa* detected by UV-Visible spectrophotometry in different media. Figure S1. Standard curve of *L-dopa* in (A) SGF, (B) SIF, and (C) PBS. Figure S2. UV-Visible spectra of *L-dopa* in (A) SGF, (B) SIF, and (C) PBS. Figure S3. Dynamic light scattering spectra of M. pruriens seed extract nanogel presenting (A) size distribution and (B) zeta potential value.
