# Supplementary material for: Development of Jelly Loaded with Nanogel Containing Natural L-Dopa from Mucuna pruriens Seed Extract for Neuroprotection in Parkinson’s Disease
Source: Pharmaceutics. 2022 May 17;14(5):1079. doi: 10.3390/pharmaceutics14051079 (PMC9147856; doi:10.3390/pharmaceutics14051079)
Supplement: Supplementary file 1 [file pharmaceutics-14-01079-s001.zip › pharmaceutics-1702022-supplementary.pdf]

Supplementary data

Table S1 LOD and LOQ of L-dopa detected by UV-Visible spectrophotometry in different media

	SGF	SIF	PBS	HPLC
LOD (μg/mL)	37.75	67.32	37.99	0.43
LOQ (μg/mL)	114.39	204.02	115.13	1.42

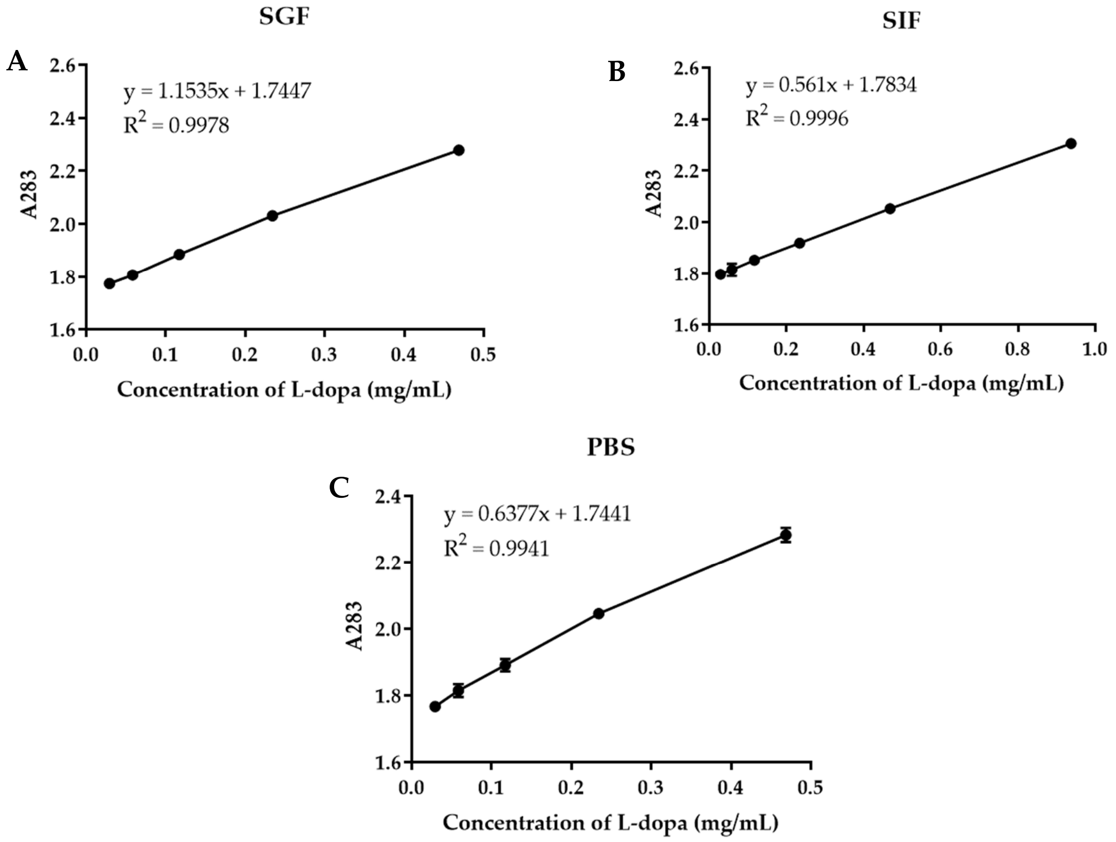

Supplementary Figure S1 Standard curve of L-dopa in (A) SGF, (B) SIF, and (C) PBS

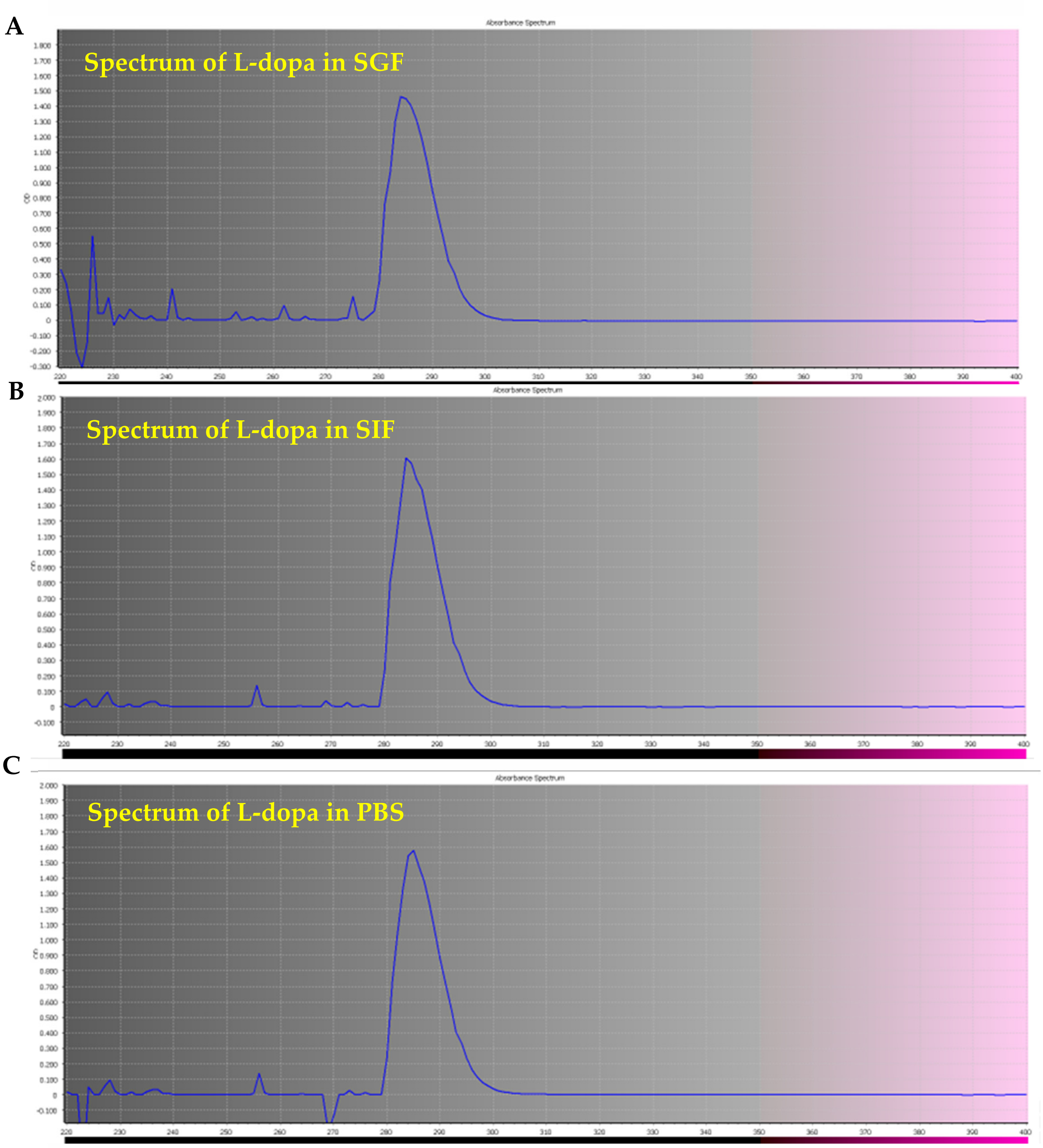

Supplementary Figure S2 UV-Visible spectra of L-dopa in (A) SGF, (B) SIF, and (C) PBS.

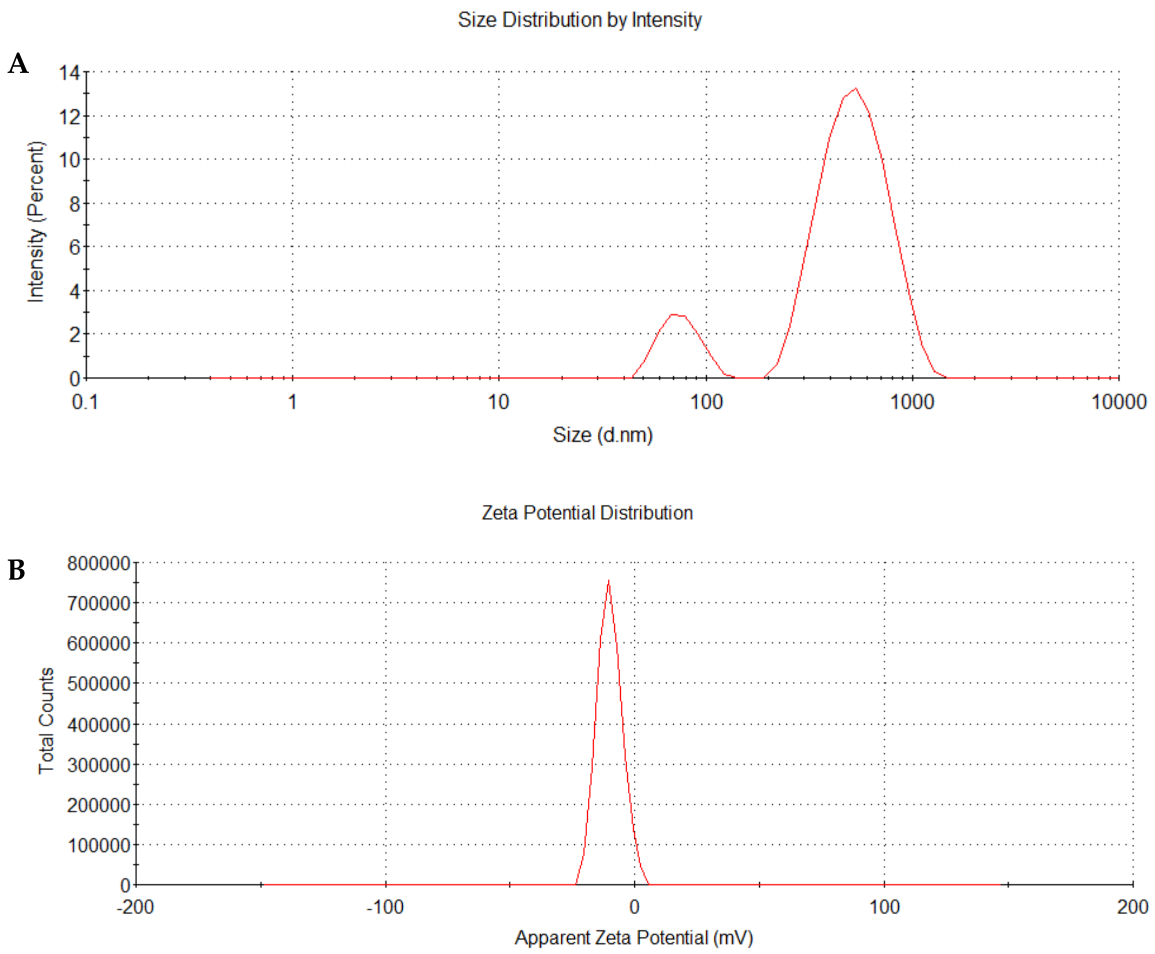

Supplementary Figure S3 Dynamic light scattering spectra of *M. pruriens* seed extract nanogel presenting (A) size distribution and (B) zeta potential value
